# Profiles of Smartphone Addiction Risk Among Middle School Students: The Roles of Childhood Neglect and Materialism Using Latent Profile Analysis, Network Analysis, and Machine Learning

**DOI:** 10.3390/ejihpe16050060

**Published:** 2026-04-24

**Authors:** Liyan Ji, Pengjin Yang, Song Zhou, Ruiyi Huang, Huimin Ma, Xiaoman Rong, Sai Zhang

**Affiliations:** 1 School of Psychology, Fujian Normal University, Fuzhou 350117, China; liyanji@fjnu.edu.cn (L.J.); qsx20240498@student.fjnu.edu.cn (P.Y.); zhousong@fjnu.edu.cn (S.Z.); qsx20240478@student.fjnu.edu.cn (R.H.); qsx20240481@student.fjnu.edu.cn (H.M.); 2 Ningboli Primary School, Tianjin 300450, China; 3 School of Public Policy, Chinese University of Hong Kong, Shenzhen 518172, China

**Keywords:** mobile phone addiction, childhood neglect, materialism, junior high school students, latent profile analysis, network analysis, machine learning

## Abstract

Using a sample of Chinese junior high school students (*N* = 1133), this study examined the latent heterogeneity and structural characteristics of mobile phone addiction risk. Latent profile analysis was conducted to identify subgroups with varying levels of risk. Network analysis was then used to model the relationships among smartphone addiction, materialism, and childhood neglect and compare structural differences across subgroups. Finally, five machine learning models were applied to model smartphone addiction scores and compare model performance across different combinations of variables. Results revealed three distinct risk groups of smartphone addiction. Network analysis indicated that loneliness-related nodes exhibited the highest expected influence in the overall network. Across latent profiles, childhood neglect-related nodes consistently occupied central positions, whereas materialism-related nodes showed relatively stable centrality. Network comparison tests further demonstrated significant structural differences across risk groups. In addition, incorporating latent profile information and centrality indices improved model performance, suggesting that these features capture individual differences in smartphone addiction. These findings provide structural evidence for the heterogeneity of mobile phone addiction risk and offer implications for subgroup-specific intervention strategies.

## 1. Introduction

With the rapid development of artificial intelligence (AI) technology, smartphones have become one of its most important application platforms, profoundly reshaping how individuals access information, engage in learning activities, and interact socially. However, the increased convenience brought by these technologies has, to some extent, also reinforced people’s dependence on smart devices. Adolescents, as one of the most frequent user groups of digital technologies, have attracted growing research attention regarding their smartphone use behaviors and potential risks. According to statistics from the China Internet Network Information Center (2025), there are approximately 154 million Internet users aged 10–19 in China, accounting for 13.7% of the total online population. Meanwhile, epidemiological studies indicate that the global prevalence of smartphone addiction among adolescents is about 25.7% (Xiong et al., 2021). These findings suggest that problematic smartphone use has become an important behavioral risk warranting attention during adolescence. Against this backdrop, identifying latent patterns of smartphone addiction risk among adolescents is of great significance for deepening our understanding of its developmental processes and for formulating more targeted prevention and intervention strategies.

### 1.1. Mobile Phone Addiction

Mobile phone addiction is commonly conceptualized as a form of behavioral addiction, characterized by core features such as salience, loss of control, mood modification, tolerance, withdrawal, conflict, and relapse (Billieux et al., 2015). Although these characteristics are primarily used to describe established addictive behaviors, related theoretical frameworks also provide important insights into the formation of mobile phone addiction risk. The Person-Affect-Cognition-Execution (I-PACE) model proposed by Brand et al. (2019) suggests that addictive behaviors arise from the dynamic interaction of personal traits, affective responses, cognitive biases, and executive functions. Therefore, individual traits play a fundamental role in explaining the risk of smartphone addiction. Based on this model, the present study focuses on two relatively stable traits closely related to adolescent development—childhood neglect and materialistic values. These traits may influence smartphone addiction risk through different psychological processes: the former primarily involves emotion regulation processes, whereas the latter is more closely associated with motivation and need compensation. Accordingly, this study integrates emotion regulation theory, compensatory Internet use theory, and self-determination theory, examining mobile phone addiction risk from the perspectives of emotion regulation, motivational formation, and basic psychological needs, in order to construct a multidimensional explanatory framework.

### 1.2. Childhood Neglect

Childhood neglect is defined by the World Health Organization as the failure of caregivers, despite having the capacity to do so, to provide for a child’s basic needs in areas such as health, education, emotional development, nutrition, and safe living conditions (World Health Organization, 2002, p. 60). As the most common and widespread form of child maltreatment (Daley et al., 2026; Topino & Gori, 2025), the harmful consequences of childhood neglect are often underestimated, with physical neglect and emotional neglect in particular being shown to have significant impacts on adolescent development. A large body of research indicates that childhood neglect leads to serious adverse outcomes in mental health (Mills et al., 2013) and impairs individuals’ emotion regulation abilities, thereby increasing their tendency to rely on external tools, such as smartphones, to cope with negative emotions (Kwak et al., 2018).

According to emotion regulation theory proposed by emotion regulation theory (Gross, 1998, 2015), individuals adopt various regulatory strategies when confronted with negative emotions. Deficits in emotion regulation resulting from childhood neglect provide an important explanatory pathway linking such experiences to the risk of mobile phone addiction. Existing research further indicates that this process operates through multiple psychological and social factors. For example, loneliness and interpersonal difficulties, as manifestations of impaired emotion regulation, have been found to mediate the relationship between childhood neglect and smartphone addiction, whereas self-control plays a moderating role in this association (Ma et al., 2020; Kwak et al., 2018). In addition, emotional intelligence has also been shown to exert a partial mediating effect (Sun et al., 2019). Taken together, childhood neglect increases the risk of smartphone addiction by undermining individuals’ emotion regulation abilities and consequently strengthening their reliance on external compensatory behaviors. Furthermore, these emotion regulation difficulties induced by neglect not only serve as a direct antecedent of addictive behaviors, but may also exert indirect effects by reshaping individuals’ value orientations, such as materialism.

### 1.3. Materialism

Materialism was defined by Belk (2001) as a value orientation in which individuals believe that material possessions are central to achieving happiness. Within the expanding context of global consumerism, this value orientation has become increasingly prevalent among adolescents and has shown a growing upward trend (Dittmar et al., 2014). According to Kardefelt-Winther’s (2014) compensatory Internet use theory, materialistic individuals are more inclined to seek emotional compensation and psychological gratification through online environments. This framework provides a theoretical basis for understanding the link between materialism and mobile phone addiction.

Within this theoretical framework, a growing body of empirical research has demonstrated a significant positive association between materialism and the risk of smartphone addiction among adolescents, operating through various compensatory psychological and behavioral mechanisms. For example, low self-esteem, as an indicator of frustrated internal needs, has been found to mediate the relationship between materialism and mobile phone addiction, whereas peer relationships moderate this compensatory pathway (Wang et al., 2020). Process-oriented and social-oriented smartphone use can be regarded as specific manifestations of compensatory use and have likewise been shown to mediate this relationship, with the mechanism differing across gender (Gentina & Rowe, 2020). In addition, fear of missing out (FoMO), reflecting unmet needs for social connectedness, has been identified as a mediator, while narcissism serves as a moderating factor that amplifies this compensatory process (Long et al., 2021). Overall, materialism promotes the development of smartphone addiction by reinforcing individuals’ reliance on external compensatory resources. However, although the association between materialism and mobile phone addiction has been well-established, the interactive mechanisms between materialism and early life experiences, such as childhood neglect, remain to be further integrated and examined.

### 1.4. Childhood Neglect and Materialism

To further explain the potential internal link between childhood neglect and materialism, self-determination theory (SDT; Deci & Ryan, 1980; Ryan & Deci, 2000) provides a critical theoretical foundation. According to this theory, relatedness, autonomy, and competence are essential basic psychological needs for individual development. Childhood neglect, particularly emotional neglect, directly undermines adolescents’ sense of relatedness (feeling cared for and supported) and further indirectly hinders the development of autonomy (feeling a sense of choice) and competence (feeling capable of effectively dealing with the environment). When these basic needs are chronically unmet, individuals are more likely to turn to external goals and material values as alternative sources of satisfaction, thereby gradually developing higher levels of materialistic orientation. On this basis, individuals may further rely on media such as smartphones to obtain recognition, a sense of control, or emotional relief, in order to compensate for the loss of self-worth caused by neglect. Through this compensatory process, childhood neglect may not only facilitate the formation of materialistic values but also, via this value orientation, further strengthen individuals’ dependence on smartphones, thereby increasing the risk of smartphone addiction. Thus, self-determination theory not only provides an important perspective for understanding the formation of materialism, but also offers a theoretical foundation for linking the full pathway from early environmental factors (childhood neglect), to value orientation bias (materialism), to maladaptive behaviors (smartphone addiction).

### 1.5. Latent Profile Analysis

In recent years, an increasing number of studies have focused on the latent heterogeneity of smartphone addiction among adolescents and have employed latent profile analysis (LPA) to classify different risk groups (Ferguson et al., 2020). Existing research has consistently identified multiple subgroups with varying levels of addiction risk, ranging from low-risk to high-risk or severe addiction profiles (Kabadayi, 2024; Williams et al., 2023; Xiong et al., 2023), collectively suggesting substantial heterogeneity in adolescent smartphone addiction. However, prior studies have primarily concentrated on general psychological and family environmental factors, while the differential roles of developmental risk factors (e.g., childhood neglect) and value-related factors (e.g., materialism) across distinct addiction risk profiles remain underexplored. Accordingly, the present study focused on junior high school students and first employed LPA to identify subgroups with varying levels of smartphone addiction risk. Subsequently, network analysis was applied to examine the structural relationships between childhood neglect and materialism, as well as their differential roles across these risk groups.

### 1.6. Network Analysis

After identifying latent profiles, the present study further introduced network analysis (NA). NA enables the examination of the statistical association structure among multiple psychological variables and facilitates a more comprehensive understanding of their interrelationships by identifying key nodes (e.g., centrality indices) (Borsboom et al., 2021; Hevey, 2018). In this study, network analysis was used to visualize the overall variable network and identify central nodes, thereby characterizing the relational structure among materialism, childhood neglect, and smartphone addiction-related variables. On this basis, the study further compares the network structural characteristics across different latent profiles to examine the positions and roles of materialism and childhood neglect within the networks of distinct smartphone addiction risk groups, thereby revealing structural differences within latent subpopulations.

### 1.7. Machine Learning

Building on the identification of latent subgroups and the analysis of their structural differences, the present study further introduces machine learning (ML) methods to examine the practical utility of latent profile information in predicting smartphone addiction risk. ML is defined as a class of algorithms that automatically learn patterns from data for prediction or decision-making (Murphy, 2012, p. 1). In psychological and behavioral sciences, ML has been widely applied to improve predictive accuracy and to conduct risk assessment at the individual level, thereby supporting screening and intervention research (Orrù et al., 2020; Bzdok et al., 2018). In this study, multiple machine learning algorithms were employed to model both observed variables and latent profile classifications, and performance metrics were used to compare different combinations of predictors and models. This approach aims to examine how latent profile information and network-based features contribute to model performance beyond traditional observed variables in representing mobile phone addiction.

In summary, within this context, the present study aims to explore the latent heterogeneity of mobile phone addiction risk among junior high school students using LPA. Building on this, NA was employed to examine the relational structure between childhood neglect and materialism across different addiction risk subgroups, and the network comparison test (NCT) was further used to compare network structural differences between these subgroups. On this basis, the study further introduces machine learning methods to predict mobile phone addiction risk and to compare the performance of different combinations of predictors and models, thereby evaluating the incremental predictive value of latent profile information.

**H1:**  *Mobile phone addiction among junior high school students exhibits latent heterogeneity.*

**H2:**  *Network structures differ across smartphone addiction risk subgroups.*

**H3:**  *The inclusion of latent profile information and network features improves the predictive performance of mobile phone addiction risk.*

## 2. Method

### 2.1. Participants

This study utilized convenience sampling and targeted seventh- and eighth-grade students (aged 12–14), a group considered representative of adolescents in the junior high school stage. A total of 1200 paper-based questionnaires were distributed, and 1167 were returned. During data screening, 34 questionnaires were excluded due to missing data or invalid response patterns (e.g., straightlining or inconsistent responses across similar items). The final sample consisted of 1133 valid responses. Among the valid participants, 575 were male (50.75%) and 558 were female (49.25%).

This study was approved by the Institutional Review Board of the first author’s university. Prior to data collection, written informed consent was obtained from all participants and their parents or legal guardians. Participants were informed of the potential risks and benefits of the study and were explicitly advised of their right to withdraw at any time. All procedures related to data collection and processing were conducted in accordance with the ethical standards outlined in the World Medical Association’s Declaration of Helsinki.

### 2.2. Measures

#### 2.2.1. Mobile Phone Addiction Index

Mobile phone addiction was assessed using the Mobile Phone Addiction Index (MPAI) developed by Leung (2008). The scale consists of 17 items measuring four dimensions: Inability to Control Craving, Feeling Anxious & Lost, Withdrawal/Escape, and Productivity Loss. Items are rated on a five-point Likert scale (1 = “Never” to 5 = “Always”), with higher total scores indicating greater tendencies toward mobile phone addiction. In the present sample, the scale demonstrated good internal consistency (Cronbach’s α = 0.92). The internal consistency coefficients for the four dimensions were 0.84 (Inability to Control Craving), 0.84 (Feeling Anxious & Lost), 0.87 (Withdrawal/Escape), and 0.75 (Productivity Loss), indicating satisfactory reliability across subscales.

#### 2.2.2. Material Values Scale

Materialistic values were assessed using the Material Values Scale (MVS) developed by Richins (2004), with reference to the Chinese translation and revision by Li and Guo (2009). The scale consists of six items measuring three core dimensions: defining success through wealth, centering life around the acquisition of material goods, and pursuing happiness through material possession. Items are rated on a five-point Likert scale (1 = “Strongly disagree,” 5 = “Strongly agree”), with higher scores reflecting stronger materialistic value orientations. In the present study, the scale demonstrated acceptable internal consistency (Cronbach’s α = 0.75).

#### 2.2.3. Measurement of Childhood Neglect

Childhood neglect was assessed using the emotional neglect and physical neglect subscales of the Chinese version of the Childhood Trauma Questionnaire-Short Form (CTQ-SF), translated and revised by Zhao et al. (2005). The original CTQ-SF, developed by Bernstein et al. (2003), consists of 28 items across five subscales: emotional neglect, physical neglect, emotional abuse, physical abuse, and sexual abuse. In the present study, only the emotional neglect and physical neglect subscales were used. Items are rated on a five-point Likert scale (1 = “Never,” 5 = “Always”). Items 2, 5, 6, 7, 8, 9, and 10 are reverse scored. Based on the scoring criteria, emotional neglect scores ≥ 15 and physical neglect scores ≥ 10 indicate the presence of the corresponding type of childhood neglect. In this study, the scale demonstrated good internal consistency (Cronbach’s α = 0.86).

### 2.3. Data Analysis

IBM SPSS 27.0 was used for preliminary data processing, descriptive statistics, reliability analyses, and inter-variable correlation analyses. To assess potential common method variance, Harman’s one-factor test was conducted by performing an unrotated principal component factor analysis on all measurement items. The results indicated a KMO value of 0.91 and a significant Bartlett’s test (*χ*^2^ = 17,864.77, *p* < 0.001). The first factor accounted for 26.60% of the total variance, which is below the 40% threshold recommended by Podsakoff et al. (2003), suggesting that common method bias was unlikely to meaningfully affect the findings. Subsequently, statistical analyses addressing the four research aims outlined in the introduction were carried out as described below.

#### 2.3.1. Latent Profile Analysis and Model Selection

LPA was conducted in Mplus 8.3 to identify subgroups of students characterized by different levels of mobile phone addiction. Robust maximum likelihood estimation (MLR) was applied to estimate a series of competing models. Model comparisons were based on multiple fit indices, including the log-likelihood (LL), Akaike information criterion (AIC), Bayesian information criterion (BIC), and adjusted BIC (adj-BIC), with lower values indicating better fit. Classification accuracy was evaluated using entropy, which ranges from 0 to 1; values above 0.80 generally indicate clear class separation and high classification precision (Lubke & Muthén, 2007). In addition, the Lo–Mendell–Rubin test (LMR) and the bootstrap likelihood ratio test (BLRT) were used to compare adjacent models (N-class model vs. (N − 1)-class model). A significant *p*-value on these tests suggests that the model with an additional class provides significantly better fit. Based on a comprehensive evaluation of all fit indices and likelihood ratio tests, the optimal latent profile model was selected for further analysis.

After establishing the optimal profile solution, subsequent analyses were conducted in SPSS 27.0 to examine associations among mobile phone addiction, childhood neglect, and materialism, as well as differences across latent profiles. Specifically, correlation analyses assessed the associations among the three variables, and one-way analysis of variance (ANOVA) was used to test whether latent profiles differed significantly on childhood neglect and materialism, thereby characterizing the psychological and behavioral features of each profile.

#### 2.3.2. Network Analysis Procedure

To better understand the nature of the three distinct latent profiles of mobile phone addiction and how childhood neglect and materialism manifest across these profiles, this study employed R 4.4.2 for data analysis. Network structures were estimated using the bootnet package (Epskamp et al., 2018) with the EBICglasso method, resulting in a regularized partial correlation network. Correlations were computed using the “cor_auto” procedure, which automatically selects the appropriate correlation type based on the measurement level of the variables. Given the ordinal Likert-type nature of the items, this procedure is designed to account for ordinal data in network estimation. In the network, each item is represented as a node, and the associations between items are represented as edges. Edges can be positive (blue) or negative (red), and the thickness of the edges indicates the strength of the associations between nodes.

To identify key nodes within the networks, four centrality indices were computed: expected influence (EI), strength, betweenness, and closeness. EI takes into account the sign of edge weights and thus provides a more comprehensive measure of node importance. In networks containing both positive and negative edges, EI provides a more appropriate measure compared to traditional centrality indices. Accordingly, nodes were ranked based on EI, and the top ten nodes were identified as core nodes. The NetworkComparisonTest package was then used to examine structural differences in network organization across the latent profiles.

To assess the robustness of the network results, nonparametric bootstrapping procedures were conducted using the bootnet package (Epskamp & Fried, 2018). First, 5000 bootstrap samples were generated to compute confidence intervals (CIs) for edge weights. Second, centrality difference tests were performed to evaluate whether differences in centrality values between nodes were statistically significant. Third, case-dropping bootstrap analyses were conducted to evaluate the stability of EI across subsamples of decreasing size (from 100% to 10%). The stability of EI was quantified using the correlation stability coefficient (CS-coefficient), which should exceed 0.25 and ideally be greater than 0.50 (Epskamp et al., 2018).

#### 2.3.3. Machine Learning Models and Training

This study developed five machine learning (ML) regression models: linear regression, ridge regression, lasso regression, elastic net regression, and extreme gradient boosting (XGBoost). Model training and testing were conducted in the R 4.4.2 environment. Linear regression assumes a linear association between predictors and the outcome variable (Seber & Lee, 2003), with parameters estimated through ordinary least squares (OLS) by minimizing the sum of squared residuals. Ridge regression (Hoerl & Kennard, 1970) extends this framework by adding an L2 regularization term to penalize the squared magnitude of coefficients, thereby reducing variance and improving model stability. Lasso regression (Tibshirani, 1996) applies L1 regularization, shrinking some coefficients to zero and thus achieving variable selection and model simplification (Friedman et al., 2010). Elastic net regression (Zou & Hastie, 2005) integrates both L1 and L2 regularization, balancing lasso’s feature selection capability with ridge’s robustness, making it suitable for high-dimensional data with multicollinearity. In contrast, XGBoost (Chen & Guestrin, 2016) is a gradient boosting framework based on decision trees; it iteratively constructs weak learners while incorporating regularization, shrinkage, and column subsampling to enhance generalization performance.

To assess generalization performance and reduce bias from random data partitioning, repeated hold-out validation was adopted. In each iteration, the dataset was randomly divided into a training set (80%) and a testing set (20%), and this process was repeated 1000 times to obtain a stable performance distribution. During training, fivefold cross-validation was conducted within the training set to improve model stability and mitigate overfitting. Final model performance was evaluated using the outer testing set.

Three metrics were used to evaluate predictive performance: mean absolute error (MAE), root mean squared error (RMSE), and the coefficient of determination (R^2^). MAE reflects the average absolute deviation between the predicted and observed values, whereas RMSE represents the square root of the average squared prediction error, placing greater weight on larger errors. Lower MAE and RMSE values indicate higher prediction accuracy. R^2^ reflects the proportion of variance explained by the model and ranges from (−∞, 1], with values closer to 1 indicating better model fit.

## 3. Result

### 3.1. Latent Profile Analysis of Junior High School Students’ MPAI

**Figure 1:**
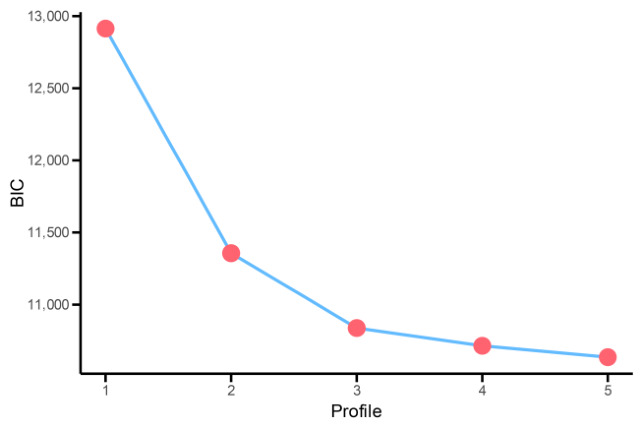
The BIC index of each latent profile model was drawn into a steep slope map.

To identify latent profiles of smartphone addiction among junior high school students, this study used standardized scores from the four dimensions of the Mobile Phone Addiction Scale to estimate latent profile models with one to five profiles (see Table 1). The results show that as the number of profiles increased, the LL, AIC, BIC, and aBIC values decreased progressively, indicating continuous improvement in model fit. In stepwise model comparisons, the LMR and BLRT tests for the two- to four-profile solutions were all statistically significant (*p* < 0.05), suggesting that adding profiles significantly improved model fit compared to the preceding model; however, when the number of profiles increased to five, the LMR test was no longer significant, indicating that the five-profile model did not yield a significant improvement over the four-profile model and was therefore not retained. Among the candidate two- to four-profile models, further comparison revealed that the three-profile model had the highest entropy value (0.84), exceeding that of the four-profile model (0.81), indicating better classification accuracy and greater certainty in profile assignment. Additionally, the decrease in BIC values became markedly smaller after the three-profile model, suggesting that further increases in the number of profiles resulted in only limited improvement in model fit (see Figure 1). Therefore, considering model fit indices, classification accuracy, and model parsimony, as well as the substantive interpretability of the profile solution, the three-profile model was ultimately selected as the optimal latent profile structure.

**Figure 2:**
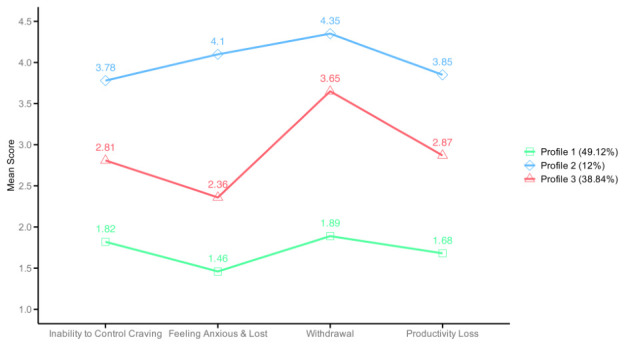
Line graph of the mean values of the three latent profiles of junior high school students for each dimension of MPAI.

To further examine differences among the three latent profiles across the four dimensions of mobile phone addiction, an analysis of variance (ANOVA) was conducted while controlling for grade. The results (see Figure 2 and Table 2) revealed a significant main effect of latent profile, *F*_(2,1129)_ = 2266.78, *p* < 0.001, η_p_^2^ = 0.80; a significant main effect of dimension, *F*_(2.63,2963.12)_ = 17.32, *p* < 0.001, η_p_^2^ = 0.02; and a significant interaction between profile and dimension, *F*_(5.25,2963.12)_ = 45.97, *p* < 0.001, η_p_^2^ = 0.08. Because the assumption of sphericity was violated, degrees of freedom were corrected using the Huynh–Feldt method.

Subsequent simple effects analyses indicated that the three latent profiles differed significantly across all four dimensions, all *F*s_(2,1130)_ > 448.48, *p*s < 0.001, η^2^ > 0.44. Post hoc pairwise comparisons using Tukey’s HSD further showed a consistent pattern across all dimensions, with scores following the order: Profile 2 > Profile 3 > Profile 1 (*p*s < 0.001). Based on these patterns, Profile 1 was labeled as the “low-risk group”, Profile 3 as the “moderate-risk group”, and Profile 2 as the “high-risk group”.

### 3.2. Correlations Between MPAI, CN, and MVS Across Latent Profiles and Gender Differences

In the total sample (*n* = 1133), Pearson correlation analyses indicated that mobile phone addiction, materialism, and childhood neglect were all significantly and positively correlated with one another (*r*s = 0.44, 0.26, and 0.17, respectively, *p*s < 0.01; see Table 3). In addition, independent-samples t tests showed that girls reported slightly higher levels of mobile phone addiction than boys; however, this difference was not statistically significant, *t*_(1131)_ = −0.63, *p* = 0.49.

**Figure 3:**
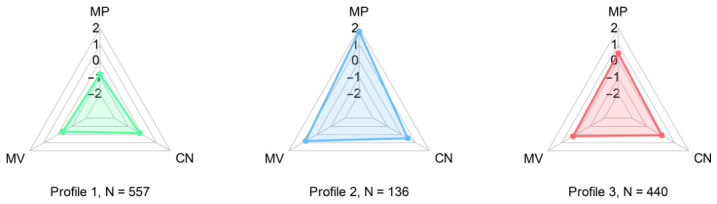
The radar charts showing the mean standardized scores for MPAI, MVS, and CN in the three latent profiles. MPAI, mobile phone addiction index, MVS, material values scale, CN, childhood neglect.

The associations among the variables differed across latent profiles (see Table 3 and Figure 3). In Profile 1 (*n* = 557), mobile phone addiction, materialism, and childhood neglect were all significantly and positively correlated with one another (*r*s = 0.27, 0.17, and 0.16, respectively, *p*s < 0.01). In Profile 2 (*n* = 136), no significant correlations were observed among the three variables (*r*s < 0.10, *p*s > 0.05). In Profile 3 (*n* = 440), mobile phone addiction was significantly and positively associated with both materialism and childhood neglect (*r*s = 0.17 and 0.18, *p*s < 0.01), whereas the association between materialism and childhood neglect was not significant (*r* = 0.06, *p* > 0.05).

To further examine the interaction between latent profiles and gender on mobile phone addiction risk, a two-way analysis of variance was conducted. The results indicated that the main effect of gender was nonsignificant, *F*_(1,1127)_ = 0.70, *p* > 0.05, and the interaction between gender and latent profile was also nonsignificant, *F*_(2,1127)_ = 0.44, *p* > 0.05. Only the main effect of latent profile was significant, *F*_(2,1127)_ = 2292.54, *p* < 0.001, η^2^ = 0.80, suggesting substantial differences in mobile phone addiction risk across latent profiles, while gender did not significantly influence these differences.

### 3.3. Network Analysis of MPAI, MVS, and CN Among Junior High School Students

#### 3.3.1. Network Analysis of the Overall MPAI Network and Three Latent Profile Networks

**Figure 4:**
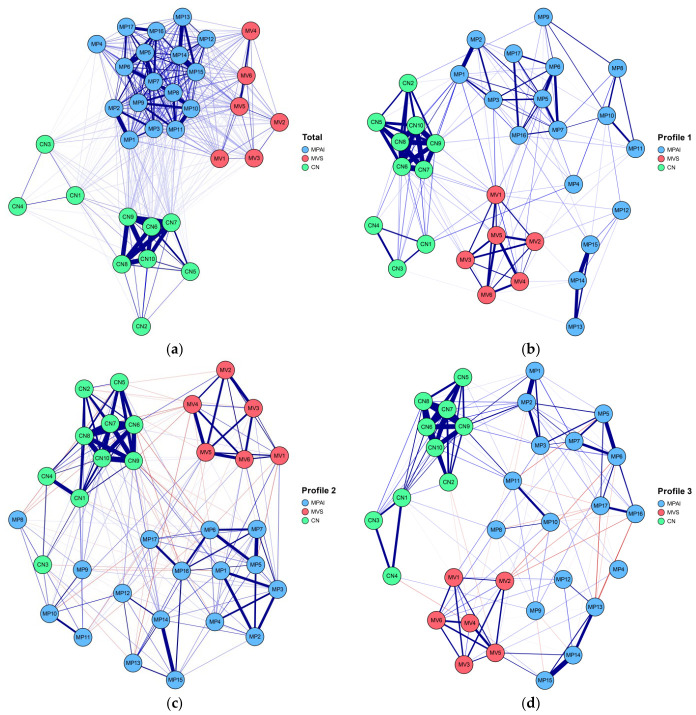
The overall and three latent profile network structures of junior high school students. The overall and three escape networks were formed by pairwise correlating 33 items from the mobile phone addiction, material values scale, and childhood neglect based on students’ responses. Each node in the network represents an item in the scale. Connections between nodes represent correlations between items. A thick line indicates that the correlation coefficient is close to 1.0, and a thin line indicates that it is close to 0. (**a**) The overall network; (**b**) the low-risk network; (**c**) the high-risk network; (**d**) the moderate-risk network.

To examine structural differences in the item-level network structures of mobile phone addiction, materialism, and childhood neglect, network models were estimated for the overall sample and for each of the three latent profiles identified through LPA. The correspondence between nodes and their respective items is provided in Table 4. The overall network contained 216 edges with non-zero weights, with an average edge weight of 0.07. As illustrated in Figure 4a, the strongest connections emerged between MP15 and MP14, MP2 and MP1, and CN7 and CN6.

Across the three latent profile networks, a total of 528 edges were generated, with 85, 51, and 63 non-zero weighted edges in Profile 1, Profile 2, and Profile 3, respectively. The average edge weights for the three profiles were 0.10, 0.13, and 0.11. As shown in Figure 4b, Profile 1 exhibited the strongest connections between CN6 and CN7, MP1 and MP2, and MP14 and MP15. In Profile 2 (Figure 4c), the strongest associations occurred between CN6 and CN7, CN1 and CN4, and CN9 and CN10. In Profile 3 (Figure 4d), the strongest connections were observed between MP14 and MP15, MP1 and MP2, and MP5 and MP6.

#### 3.3.2. Indicators of Centrality for the Overall Network and the Three Latent Profile Networks

**Figure 5:**
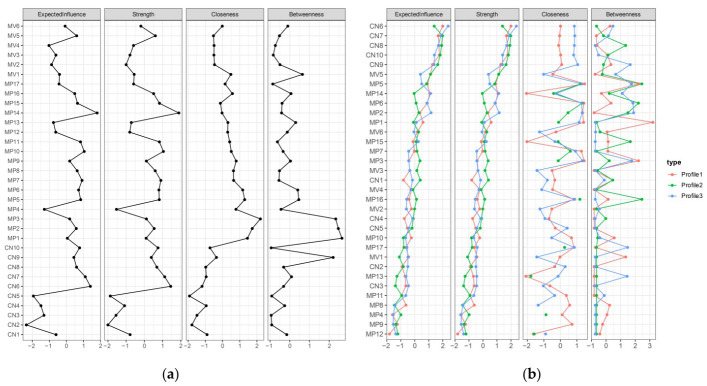
Summary of structural centrality analysis indicators for the overall and three latent profile networks. (**a**) the overall network; (**b**) the three latent profile networks.

The EI indices for the overall network and the three latent profile item networks are presented in Figure 5. In the overall network, the ten nodes with the highest EI values, listed in descending order, were MP14, CN6, CN7, MP10, MP7, MP5, MP11, CN10, MP6, and MP15, indicating that these nodes exerted the greatest influence within the network.

Across the three latent profile networks, the top ten nodes ranked by EI also varied notably by profile. For Profile 1, the highest EI values were observed for CN6, CN10, CN8, CN7, CN9, MV5, MP14, MP5, MP6, and MP1. For Profile 2, the top nodes were CN7, CN8, CN10, CN9, CN6, MV5, MP5, CN1, MP3, and MP2. For Profile 3, the highest-ranked nodes were CN6, CN7, CN8, CN10, CN9, followed by MP2, MP14, MP6, MP5, and MV5.

#### 3.3.3. Accuracy and Stability of the Overall Network and the Three Latent Profile Networks

The bootstrapped edge-weight estimates for the overall network and the three latent profile networks (see Supplementary Figure S1a–d), the node centrality difference tests (see Supplementary Figure S2a–d), and the stability estimates of the centrality metrics (see Supplementary Figure S3a–d) indicated that all networks demonstrated adequate accuracy. The correlation stability (CS) coefficient for the overall network was 0.75, reflecting high stability. Similarly, Profile 1 and Profile 3 showed strong stability, each with a CS coefficient of 0.75. In contrast, Profile 2 exhibited a lower CS coefficient (0.36), indicating comparatively reduced stability. Although this value falls below the optimal benchmark of 0.50, it remains above the minimum acceptable threshold of 0.25 (Epskamp et al., 2018), suggesting that the network retains interpretive validity.

#### 3.3.4. Comparison of the Three Latent Profile Networks

Global and local network invariance tests were conducted to compare the item networks across the three latent profiles. The network structure invariance test and global strength invariance test indicated significant differences only between Profile 1 and Profile 2 (M_c1–c2_ = 0.34, p_c1–c2_ = 0.02; S_c1–c2_ = 1.83, p_c1–c2_ = 0.03). No significant differences were observed between Profile 1 and Profile 3, nor between Profile 2 and Profile 3 (M_c1–c3_ = 0.18, p_c1–c3_ = 0.31; S_c1–c3_ = 1.45, p_c1–c3_ = 0.19, M_c2–c3_ = 0.26, p_c2–c3_ = 0.55; S_c2–c3_ = 0.38, p_c2–c3_ = 0.10).

The edge invariance test further revealed that each pair of latent profile networks differed in specific edge weights. Specifically, four edges differed significantly between Profile 1 and Profile 2: MP1–MP2, MP5–MP7, MP13–MP14, and MP6–MP16 (*p*s < 0.05). Two edges differed significantly between Profile 1 and Profile 3: MP13–MP14 and MP13–MP17 (*p* = 0.04, *p* = 0.01). Between Profile 2 and Profile 3, two edges showed significant differences: MP1–MP3 and MP5–MP7 (*p* = 0.004, *p* = 0.001).

### 3.4. Machine Learning of MPAI, MVS, and CN Among Junior High School Students

This study conducted three sets of prediction tasks, and the performance of the five ML models is presented in Table 5. In the first prediction task, when using item-level scores of the MVS and CN scales as predictors, ridge regression showed the best performance, with the lowest RMSE and the highest R^2^. In the second prediction task, the EI values derived from the overall network were used to weight the item scores of MVS, CN, and MPAI, and XGBoost demonstrated the best performance when MVS and CN were included as predictors, achieving the lowest RMSE, the highest R^2^, and an MAE close to the minimum value. In the third prediction task, after incorporating the latent class variable identified through LPA into the predictor set, lasso regression yielded the best overall performance, achieving optimal results across all three-evaluation metrics (RMSE, MAE, and R^2^).

## 4. Discussion

Overall, the findings of this study indicate that mobile phone addiction risk among junior high school students exhibits pronounced heterogeneity, involving the joint influence of multiple factors, including emotion regulation, early family support, and behavioral control. By integrating LPA, NA, and ML approaches, the present study examined this heterogeneity from three perspectives: subgroup identification, structural relationships, and model comparison.

### 4.1. Heterogeneity of Mobile Phone Addiction Risk

First, the results of the LPA indicated that smartphone addiction risk among junior high school students does not follow a single continuous distribution, but can be categorized into three distinct groups—low-, moderate-, and high-risk—thus supporting Hypothesis 1. This finding suggests that individuals may exhibit qualitatively different risk profiles, rather than merely varying in the severity of risk. Further analyses showed that although female students had slightly higher smartphone addiction scores than male students, the difference was not statistically significant, indicating that individual differences are more likely attributable to latent class membership rather than gender. In addition, overall correlation analyses revealed significant positive associations among mobile phone addiction, materialism, and childhood neglect. However, subgroup analyses demonstrated that these associations were not consistent across latent profiles, suggesting that the roles of these psychosocial factors may differ across risk groups.

### 4.2. Structural Characteristics: Evidence from Network Analysis

Building on these findings, NA further revealed the structural characteristics within different risk groups. The centrality results of the overall network indicated that emotion-dependence nodes (MP14, MP15), anxiety and loss-related nodes (MP10, MP11), and loss-of-control use nodes (MP7, MP5, MP6) in the smartphone addiction scale occupied central positions. At the same time, family support-related nodes (CN6, CN7, CN10) in the childhood neglect scale also demonstrated relatively high centrality. These findings suggest that smartphone addiction risk may be closely associated with adolescents’ emotion regulation processes, and that a lack of early family support may influence emotional experiences and regulation strategies, thereby increasing the tendency to use smartphones for emotional relief (Brand et al., 2019). Notably, MP14 (“When you feel lonely, do you use your phone to make yourself feel better?”) exhibited the highest centrality, further indicating that smartphone use may, to some extent, be related to coping with loneliness and negative emotions. This finding is consistent with prior research on emotional avoidance and compensatory smartphone use (Elhai et al., 2017).

Further examination of the latent profile-specific networks revealed that different risk groups exhibited both commonalities and notable differences in their structural characteristics. The shared pattern was reflected in the consistent centrality of childhood neglect-related nodes (CN6–CN10) across all profiles, suggesting that a lack of family support may represent a common background factor across risk groups.

In terms of differences, the emotion-dependence node MP14 occupied a central position in the low- and moderate-risk groups but was markedly weakened in the high-risk group. In contrast, loss-of-control use nodes (MP5, MP6, MP1, MP2, MP3) remained central within the high-risk group. This pattern reflects a differentiated distribution of emotion-related and behavior-related features across risk groups and is consistent with the distinction between “emotion-driven” and “behavior-reinforcement” patterns. Specifically, mobile phone use in the low-risk group appears to be more closely associated with coping with negative emotions, whereas the moderate-risk group shows a coexistence of emotional and behavioral characteristics, and the high-risk group is primarily characterized by behavioral dysregulation.

In addition, the extrinsic goal orientation node (MV5) maintained relatively stable centrality across all profiles, suggesting that some individuals, in the context of insufficient family support, may seek compensation through extrinsic values and use mobile phones as a means of obtaining emotional or social gratification. This finding is consistent with the perspective of self-determination theory (Ryan & Deci, 2000).

### 4.3. Differences Across Risk Profiles

The results of the NCT partially supported Hypothesis 2, indicating that the network structures across different latent profiles were not entirely identical. Specifically, a significant difference was observed between the low- and high-risk groups, whereas the moderate-risk group did not differ significantly from either of the other two groups, suggesting a certain degree of structural overlap. From a structural perspective, these findings further indicate that different risk groups may be characterized by distinct addiction-related features and highlight the importance of considering group differences when understanding smartphone addiction risk.

### 4.4. Model Comparison: The Role of Profile and Network Features

Finally, the machine learning results supported Hypothesis 3. In this study, predictive performance was used as an evaluation metric to compare different feature combinations and models, rather than serving solely as a prediction task. The results showed that model performance was significantly improved when features weighted by expected influence (EI) derived from the overall network were incorporated, along with classification variables obtained from latent profile analysis (LPA). These findings suggest that latent profile information and network-based features provide additional information beyond traditional observed variables, thereby demonstrating their incremental predictive value and helping to capture individual differences in mobile phone addiction risk that are not fully reflected by conventional measures. This finding is consistent with the observed heterogeneity in mobile phone addiction risk and provides a reference for future research exploring differentiated intervention strategies based on latent subgroups.

### 4.5. Theoretical Implications

From the perspectives of latent class membership and network structure, the present study elucidates the effects of childhood neglect and materialism on the risk of mobile phone addiction among middle school students, thereby providing new empirical support for the I-PACE model (Brand et al., 2019), emotion regulation theory (Gross, 1998, 2015), the Internet compensation theory (Kardefelt-Winther, 2014), and self-determination theory (Ryan & Deci, 2000). The findings indicate that a lack of early familial support and a materialistic orientation may jointly increase adolescents’ dependence on mobile phone use by undermining emotion regulation capacities and strengthening compensatory motives, thereby persistently elevating addiction risk. By integrating key theoretical propositions concerning impaired emotion regulation, frustration of psychological needs, and compensatory gratification, these results deepen our understanding of the developmental processes underlying adolescent mobile phone addiction risk and offer novel insights for refining comprehensive theoretical frameworks of behavioral addiction.

### 4.6. Practical Implications

Based on the heterogeneity in mobile phone addiction risk identified in this study, intervention strategies may benefit from a stratified and profile-based approach. Specifically, for adolescents at low risk of mobile phone addiction, interventions may focus on emotion regulation training to reduce emotionally driven smartphone use. For those at moderate risk, intervention strategies could integrate both emotion regulation and behavioral control components to mitigate the interaction between emotional avoidance and maladaptive use. For high-risk adolescents, greater attention may be given to behavioral dysregulation, with interventions oriented toward habit modification and crisis management, alongside professional support when necessary. In addition, considering the role of extrinsic goal orientation identified in this study, future interventions may incorporate activities grounded in self-determination theory to reduce the sustaining influence of extrinsic goals on addiction-related behaviors.

### 4.7. Limitations

Although this study revealed the structural relationships among childhood neglect, materialism, and mobile phone addiction, several limitations should be acknowledged. First, the use of convenience sampling may limit the representativeness and external validity of the findings; therefore, future studies should adopt multi-center probability sampling to enhance generalizability. Second, the cross-sectional design precludes the establishment of causal relationships, and the reliance on self-report measures may introduce social desirability or recall bias. Future research employing longitudinal designs and incorporating multi-source objective indicators would strengthen the reliability of these associations. Third, the unequal distribution of sample sizes across latent profile groups resulted in reduced network stability within the high-risk group. Although the stability remained within acceptable thresholds, this limitation may reduce statistical power and should be further examined in larger and more balanced samples. Finally, the machine learning approach in this study was primarily used for model comparison rather than for prospective prediction. In addition, because some predictors (e.g., latent profile membership) were derived from the same construct as the outcome variable, the interpretability of this analysis is constrained and should be understood as reflecting concurrent associations rather than independent predictive relationships. Future research could further evaluate model performance and generalizability using independent samples.

## 5. Conclusions

In conclusion, this study further advances our understanding of the heterogeneity underlying mobile phone addiction risk among adolescents. The findings indicate that distinct risk groups exhibit significant differences in behavioral characteristics: adolescents in the low- and moderate-risk groups primarily display mobile phone use patterns related to emotion regulation, whereas those in the high-risk group are more characterized by compulsive and habitual use. These differences reflect a differentiated distribution of emotion-related and behavior-related features across risk groups and are consistent with the distinction between “emotion-driven” and “behavior-reinforcement” patterns. Given the cross-sectional nature of the present study, these findings represent a possible interpretation and do not allow for inferences regarding temporal sequence or developmental processes. Future research should employ longitudinal designs and multi-source assessments to further examine these patterns and explore the associations among different risk characteristics, thereby providing a more robust empirical basis for precision prevention and stratified intervention strategies targeting adolescent mobile phone addiction risk.

## Figures and Tables

**Table 1:** Fit index of the latent profile models of mobile phone addiction in junior high school students (*n* = 1133).

Profiles Tested	LL	AIC	BIC	adj-BIC	Entropy	LMR *p*-Value	BLRT *p*-Value	Profile Probability (%)
1	−6428.63	12,873.26	12,913.52	12,888.11	-	-	-	1
2	−5632.68	11,291.36	11,356.79	11,315.50	0.83	<0.001	<0.001	62.67/37.34
**3**	**−5355.90**	**10,747.90**	**10,838.39**	**10,781.21**	**0.84**	**<0.001**	**<0.001**	**49.16/12.00/38.84**
4	−5276.56	10,599.12	10,714.87	10,641.82	0.81	<0.05	<0.001	45.98/23.12/21.89/9.00
5	−5219.44	10,494.89	10,635.80	10,546.87	0.81	>0.05	<0.001	11.47/43.43/13.24/23.65/8.21

Note. LL, likelihood ratio Chi-squared test; AIC, Akaike information criterion; BIC, Bayesian information criterion; adj-BIC, adjusted Bayesian information criterion; LMR, Lo–Mendell–Rubin likelihood ratio test; BLRT, bootstrapped likelihood ratio test. Bold text indicates the selected model.

**Table 2:** Descriptive statistics and ANOVA results for MPAI dimensions across three latent profiles (*n* = 1133).

	Profile 1 (49.12%) Low-Risk	Profile 2 (12%) High-Risk	Profile 3 (38.84%) Moderate-Risk	*F*_(2,1130)_	*p*	η^2^
Inability to Control Craving	1.81 ± 0.56 ^a^	3.79 ± 0.75 ^b^	2.81 ± 0.70 ^c^	640.37	<0.001	0.53
Feeling Anxious & Lost	1.46 ± 0.43 ^a^	4.11 ± 0.64 ^b^	2.36 ± 0.63 ^c^	1347.44	<0.001	0.71
Withdrawal/Escape	1.88 ± 0.82 ^a^	4.36 ± 0.78 ^b^	3.65 ± 0.96 ^c^	736.80	<0.001	0.57
Productivity Loss	1.68 ± 0.71 ^a^	3.86 ± 1.04 ^b^	2.87 ± 0.99 ^c^	448.48	<0.001	0.44

Note. M = mean; SD = standard deviation. Different superscript letters (^a^, ^b^, ^c^) within the same row indicate significant differences between profiles (*p* < 0.001) based on Tukey’s HSD post hoc tests. The values in parentheses indicate the proportion of each group to the total.

**Table 3:** Correlation analysis of mobile phone addiction, material values scale, and childhood neglect among junior high school students in different latent profiles.

Group	Variables	M ± SD	Mobile Phone Addiction	Material Values Scale	Childhood Neglect
Total sample (*n* = 1133)	1	2.42 ± 0.88	-	0.44 **	0.26 **
	2	2.87 ± 0.85		-	0.17 **
	3	1.92 ± 0.81			-
Low-Risk (*n* = 557)	1	1.71 ± 0.37	-	0.27 **	0.17 **
	2	2.56 ± 0.78		-	0.16 **
	3	1.76 ± 0.78			-
Moderate-Risk (*n* = 440)	1	2.83 ± 0.39	-	0.17 **	0.18 **
	2	3.04 ± 0.76		-	0.06
	3	1.99 ± 0.78			-
High-Risk (*n* = 136)	1	3.99 ± 0.46	-	0.06	0.78
	2	3.55 ± 0.86		-	0.72
	3	2.28 ± 0.88			-

Note. ** *p* < 0.01.

**Table 4:** Overview of network nodes including corresponding scale, item description, and mean (SD).

Nodes	Scale	Item Description	M	SD
MP1	MPAI	Others complain	3.04	1.28
MP2	MPAI	Told overuse	2.81	1.39
MP3	MPAI	Hide usage	2.12	1.30
MP4	MPAI	Costly bills	1.51	0.99
MP5	MPAI	Use too long	2.76	1.34
MP6	MPAI	Fail to reduce use	2.48	1.36
MP7	MPAI	Not enough use time	2.32	1.37
MP8	MPAI	Worry missed calls	2.01	1.26
MP9	MPAI	Can’t switch off	1.99	1.31
MP10	MPAI	Anxious when offline or unchecked	1.97	1.27
MP11	MPAI	Feel lost without phone access	1.80	1.18
MP12	MPAI	Unreachable without phone	2.87	1.47
MP13	MPAI	Phone talk when isolated	2.61	1.50
MP14	MPAI	Phone talk when lonely	3.00	1.48
MP15	MPAI	Phone use feel better	2.99	1.47
MP16	MPAI	Phone use disrupts tasks	2.38	1.29
MP17	MPAI	Productivity drops due to phone	2.42	1.31
MV1	MVS	Admire wealthy lifestyles	2.41	1.26
MV2	MVS	Belongings show success	3.33	1.26
MV3	MVS	Buying brings pleasure	2.76	1.23
MV4	MVS	Prefer luxury living	3.19	1.35
MV5	MVS	Want things for better life	3.11	1.31
MV6	MVS	Happier with more to buy	2.41	1.23
CN1	CN	Not enough to eat	1.46	0.93
CN2	CN	Felt cared for	2.34	1.48
CN3	CN	Parents too drunk or high	1.19	0.66
CN4	CN	Wore dirty clothes	1.34	0.91
CN5	CN	Felt loved	2.35	1.49
CN6	CN	Family looked out for me	2.13	1.30
CN7	CN	Family felt close	2.14	1.28
CN8	CN	Cared for my health	1.92	1.27
CN9	CN	Family gave support	2.24	1.39
CN10	CN	Felt important to family	2.04	1.29

Note. MPAI, mobile phone addiction index; MVS, material values scale; CN, childhood neglect.

**Table 5:** Performance of machine learning models in predicting MPAI (weighted) total scores.

Target Variable	Predictor(s)	Model	RMSE	R^2^	MAE
			Mean [95% CI]		
MPAI Total Score	MVS Item Scores	LM	9.767 [9.023, 10.573]	0.238 [0.147, 0.326]	7.755 [7.203, 8.339]
	CN Item Scores	Ridge Regression	**9.750 [8.969, 10.608]**	**0.241 [0.150, 0.336]**	7.775 [7.247, 8.408]
		Lasso Regression	9.761 [8.971, 10.625]	0.239 [0.148, 0.334]	7.768 [7.216, 8.425]
		Elastic Net Regression	9.754 [8.985, 10.585]	0.240 [0.150, 0.340]	7.770 [7.202, 8.353]
		XGBoost	9.799 [8.991, 10.654]	0.235 [0.136, 0.330]	**7.738 [7.152, 8.340]**
MPAI Weighted Total Score	MVS Weighted Item Scores	LM	4.649 [4.275, 5.027]	0.187 [0.093, 0.281]	3.694 [3.414, 3.976]
	CN Weighted Item Scores	Ridge Regression	4.642 [4.288, 4.992]	0.188 [0.106, 0.288]	3.699 [3.451, 3.946]
		Lasso Regression	4.623 [4.288, 4.992]	0.192 [0.101, 0.283]	**3.678 [3.443, 3.965]**
		Elastic Net Regression	4.636 [4.291, 4.986]	0.189 [0.099, 0.286]	3.694 [3.445, 3.941]
		XGBoost	**4.618 [4.275, 4.968]**	**0.197 [0.115, 0.292]**	3.685 [3.430, 3.946]
MPAI Weighted Total Score	MVS Weighted Item Scores	LM	2.873 [2.632, 3.115]	0.688 [0.627, 0.742]	2.295 [2.102, 2.509]
	CN Weighted Item Scores	Ridge Regression	2.876 [2.629, 3.121]	0.689 [0.628, 0.742]	2.298 [2.112, 2.502]
	LPA Profile	Lasso Regression	**2.850 [2.643, 3.083]**	**0.694 [0.643, 0.744]**	**2.282 [2.101, 2.471]**
		Elastic Net Regression	2.856 [2.616, 3.090]	0.694 [0.635, 0.748]	2.290 [2.103, 2.490]
		XGBoost	2.858 [2.641, 3.081]	0.691 [0.637, 0.745]	2.291 [2.103, 2.484]

Note. The best performance for each metric is shown in bold. RMSE, root mean squared error; R^2^, coefficient of determination; MAE, mean absolute error; MPAI, mobile phone addiction index; MVS, material values scale; CN, childhood neglect, XGBoost extreme gradient boosting, LM linear regression.

## Data Availability

The data presented in this study are available on request from the corresponding authors.

## Supplementary Materials

The following supporting information can be downloaded at: https://www.mdpi.com/article/10.3390/ejihpe16050060/s1, Figure S1: Centrality difference test results for the nodes of the networks; Figure S2: Bootstrap confidence intervals for edge weights in networks; Figure S3: Case-dropping bootstrap stability plots for the networks.
