# Supplementary material for: Profiles of Smartphone Addiction Risk Among Middle School Students: The Roles of Childhood Neglect and Materialism Using Latent Profile Analysis, Network Analysis, and Machine Learning
Source: Eur J Investig Health Psychol Educ. 2026 Apr 24;16(5):60. doi: 10.3390/ejihpe16050060 (PMC13206078; doi:10.3390/ejihpe16050060)
Supplement: Supplementary file 1 [file ejihpe-16-00060-s001.zip › ejihpe-4217658-supplementary.pdf]

Supplementary Materials

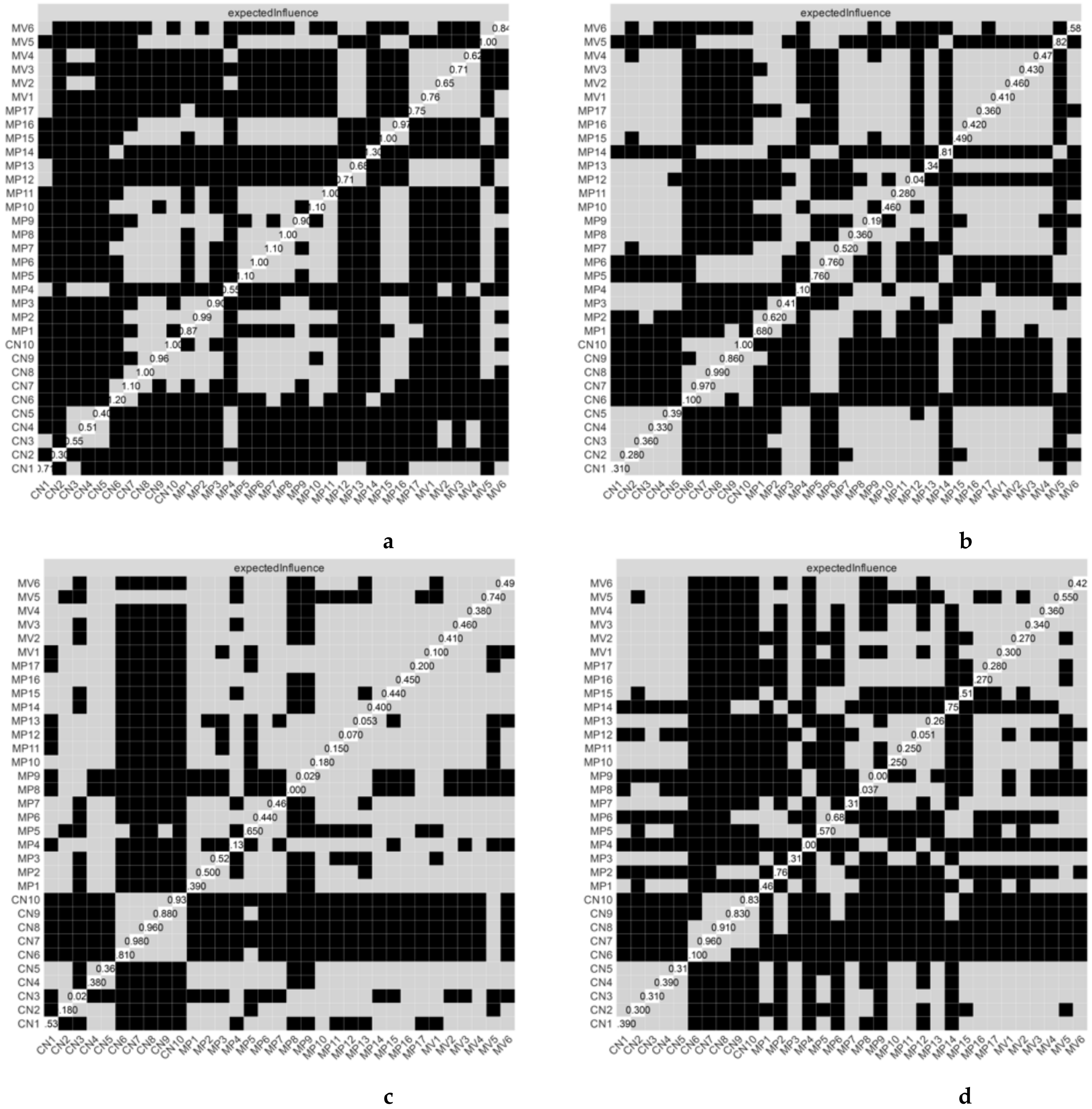

Figure S1. Centrality difference test results for the nodes of the networks.

Note. **(a)** The overall network; **(b)** the low-risk network; **(c)** the high-risk network; **(d)** the moderate-risk network. Black boxes indicate significant differences between two nodes (α = 0.05).

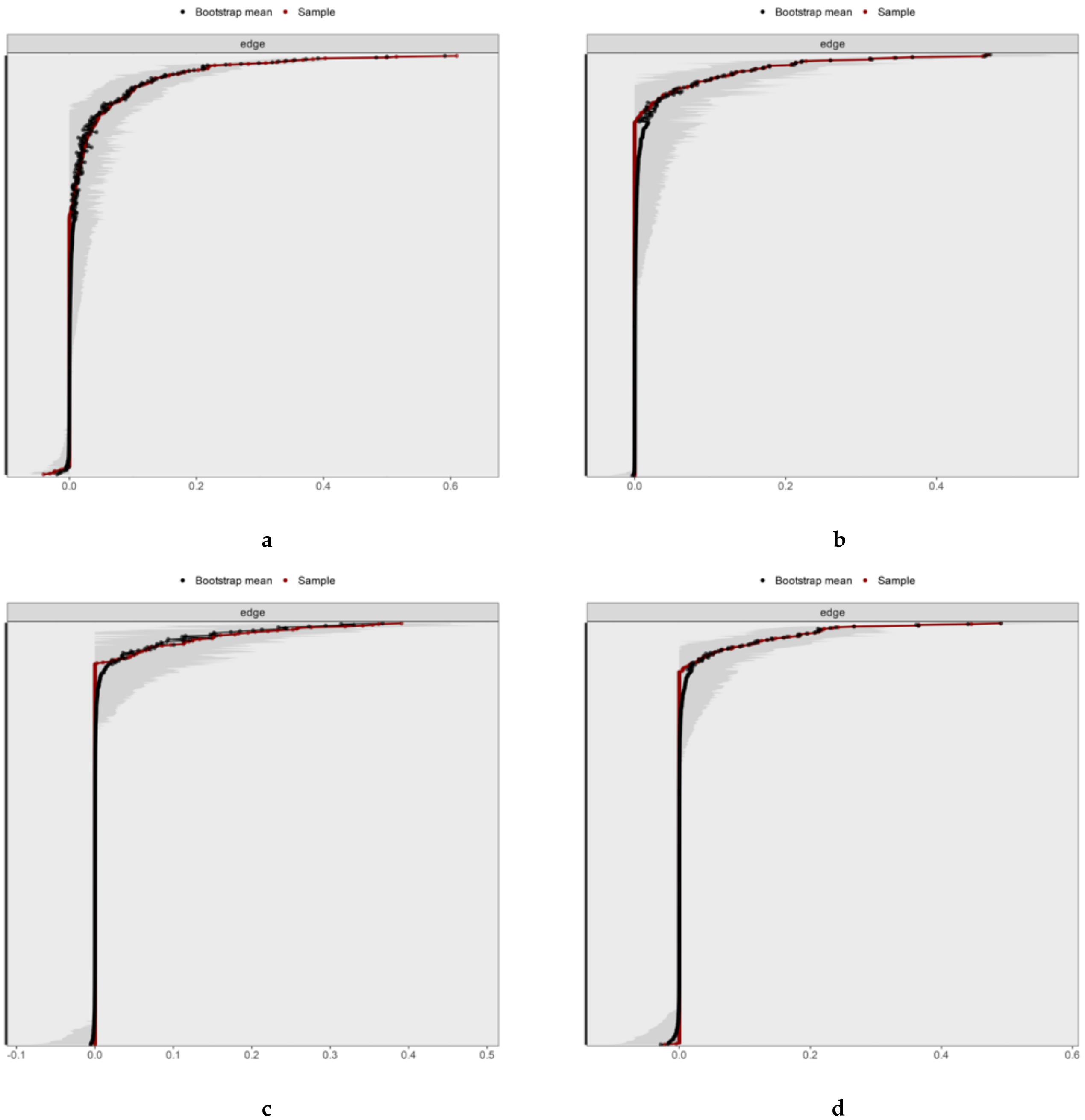

Figure. S2. Bootstrap confidence intervals for edge weights in networks.

Note. **(a)** The overall network; **(b)** the low-risk network; **(c)** the high-risk network; **(d)** the moderate-risk network. Red dots indicate sample values, black dots indicate values for each edge weight, and grey areas indicate 95% confidence intervals.

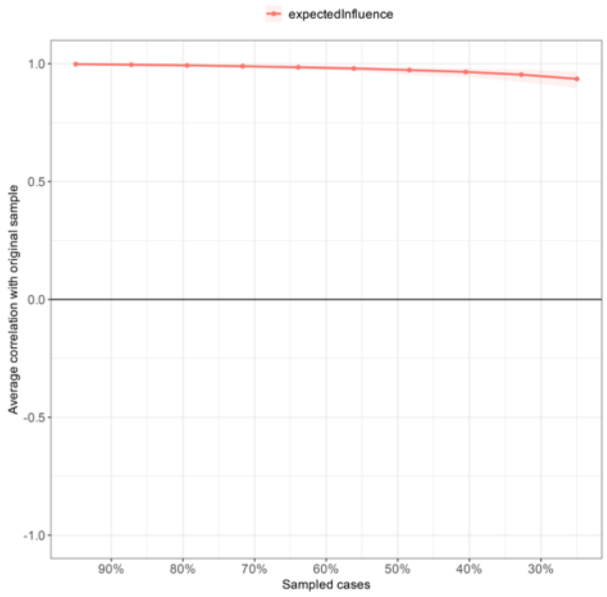

a

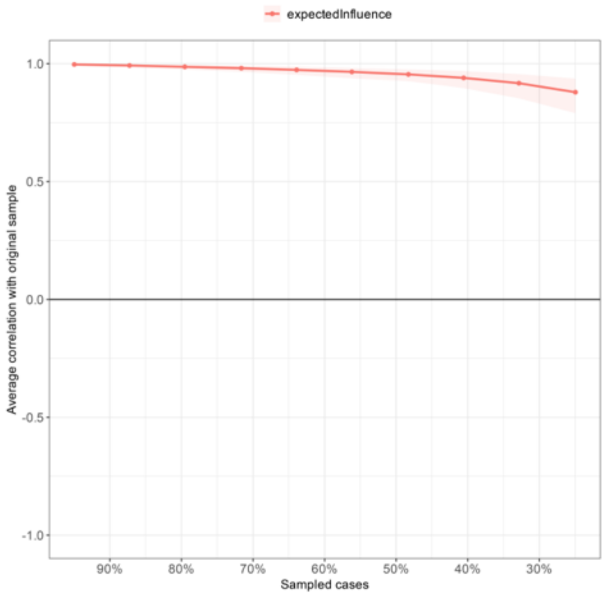

b

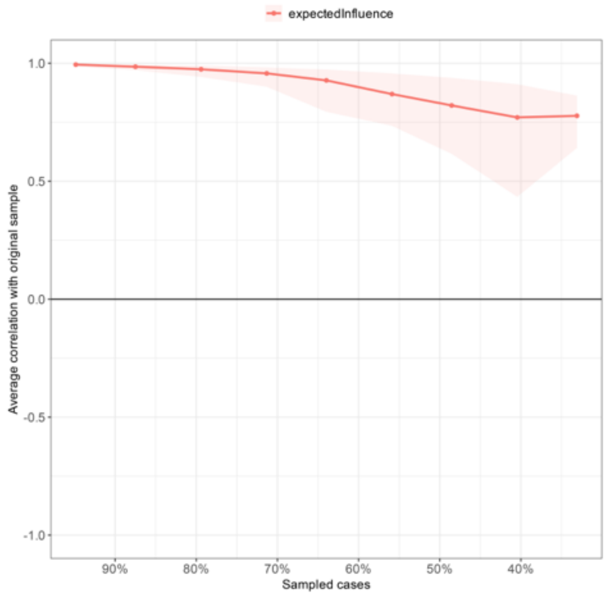

c

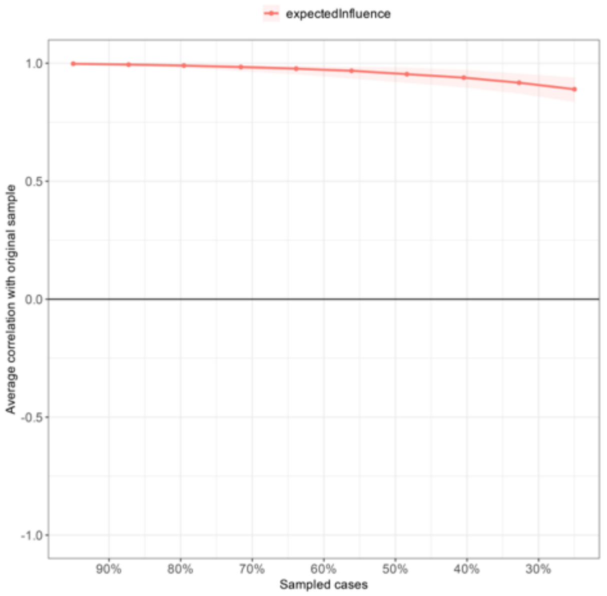

d

Figure. S3. Case-dropping bootstrap stability plots for the networks.

Note. **(a)** The overall network; **(b)** the low-risk network; **(c)** the high-risk network; **(d)** the moderate-risk network. Lines represent the average relationship between the original sample centrality and the subsamples. Shaded areas indicate the interquartile range (IQR).
